# Development and validation of a machine-learning-based triage model incorporating systemic inflammatory, nutritional, coagulation, and tumor marker parameters for identifying baseline distant metastasis in lung cancer

**DOI:** 10.3389/fonc.2026.1929804

**Published:** 2026-09-08

**Authors:** Huayue Yuan, Jiejian Chen, Wen Liu, Nianzhang Luo, Xisheng Fang

**Affiliations:** 1 Guangzhou First People’s Hospital, Guangzhou, China; 2 Guangzhou Medical University, Guangzhou, China; 3 Guangdong Medical University, Zhanjiang, China

**Keywords:** baseline risk stratification, coagulation function, distant metastasis, lung cancer, machine learning, nutritional status, predictive model, systemic inflammation

## Abstract

**Purpose:**

This study aimed to develop and validate a machine learning-based model to identify baseline distant metastasis in lung cancer by integrating multidimensional pretreatment indicators—including systemic inflammatory, tumor, coagulation, and nutritional parameters.

**Methods:**

This retrospective case–control study enrolled 408 treatment naïve patients with newly diagnosed lung cancer at Guangzhou First People’s Hospital between November 2023 and October 2024, including 202 cases with and 206 without distant metastasis. A total of 18 peripheral blood biomarkers—encompassing systemic inflammation, tumor burden, coagulation, and nutritional status—along with six demographic and clinical characteristics, were collected as candidate predictors. After initial univariate logistic regression screening, nested 5-fold cross-validation was implemented within the training set, within each fold of which the three complementary feature selection algorithms (LASSO, RFE, and Boruta) were independently applied in combination to derive a robust set of predictive features. Based on the selected features, nine machine learning classifiers were then developed within each fold, and algorithm comparison as well as optimal classifier selection were performed using the mean AUC across the five folds. Model performance was evaluated using the area under the receiver operating characteristic curve (AUC), accuracy, positive predictive value, negative predictive value, F1 score, and 95% confidence interval. The optimal model was selected based on overall performance; its calibration was assessed using calibration plots and the Hosmer–Lemeshow goodness of fit test, while clinical net benefit was quantified through decision curve and clinical impact curves. To facilitate bedside risk stratification, a nomogram was constructed based on the final model to enable individualized probability estimation. Feature importance and model interpretability were further examined using SHAP values.

**Results:**

The AdaBoost algorithm demonstrated the best performance for identifying baseline distant metastasis in lung cancer, achieving a test AUC of 0.840 (95% CI: 0.757–0.912), and was selected as the final model. A rigorous nested 5-fold cross-validation provided an unbiased AUC estimate of 0.82, which aligns closely with the test AUC of 0.84 and further confirms the model’s stability. Learning curves, decision curve analysis, and calibration curves collectively confirmed its favorable generalization, clinical net benefit, and calibration accuracy, with positive net benefit across a wide range of threshold probabilities. SHAP analysis identified CEA, SII, smoking history, CYFRA21-1, D-dimer, fibrinogen, AGR, histological type, SIRI, and NSE as the top 10 contributors to the model’s risk stratification—rankings that closely aligned with those from the decision tree model—underscoring their biological and clinical relevance.

**Conclusion:**

This study developed and internally validated an AdaBoost-based model for pretreatment identification of baseline distant metastasis in lung cancer. As a low-cost, non-invasive tool integrating routine laboratory parameters, it shows preliminary promise for early risk stratification, although external validation is required before clinical implementation.

## Background

Lung cancer remains the leading cause of cancer-related morbidity and mortality worldwide, characterized by a poor prognosis and posing a significant threat to human health. According to 2022 global cancer statistics, lung cancer accounted for 12.34% of all new malignant tumor cases and 21.36% of cancer-related deaths (1). In clinical practice, approximately 75% of patients with lung cancer present with metastasis at the time of diagnosis, with common metastatic sites including the Brain, Bone, Liver, and Adrenal glands (2). Local recurrence and distant metastasis are major contributors to the substantially increased risk of death in lung cancer patients, and more than 90% of tumor-related deaths are attributed to metastatic disease rather than the primary tumor itself (3).

Lung cancer metastasis is a multifactorial process involving systemic inflammation, immunonutrition, coagulation–fibrinolysis dysregulation and tumor-derived biomarkers (3, 4–6). Chronic inflammation plays a pivotal role in shaping the tumor microenvironment through the secretion of cytokines, chemokines, and other mediators, thereby directly promoting epithelial–mesenchymal transition (EMT) and tumor cell invasion (7). Inflammatory cells, including neutrophils, lymphocytes, platelets, and monocytes, have also been implicated in facilitating cancer dissemination and metastasis (8). A range of inflammation-based indices derived from these cellular components—such as the Neutrophil-to-lymphocyte ratio (NLR), Platelet-to-lymphocyte ratio (PLR), Lymphocyte-to-monocyte ratio (LMR), Systemic immune-inflammation index (SII), and Systemic inflammation response index (SIRI)—have been extensively investigated and validated across various solid tumors (9–11). Notably, these indices have demonstrated independent prognostic value in patients with distant metastatic non-small cell lung cancer treated with nivolumab (4) (4) (9–11). Secondly, Malnutrition further compromises immune function and promotes tumor proliferation. Decreased serum albumin levels reflect both nutritional deficiency and systemic inflammation, while elevated globulin levels are associated with tumor progression and immunosuppression (12, 13). Accordingly, composite nutritional–inflammatory markers—including the Albumin-to-globulin ratio (AGR) and Prognostic nutritional index (PNI)—have been consistently identified as independent prognostic predictors in advanced non-small cell lung cancer (14, 15), Similarly, the Hemoglobin, Albumin, Lymphocyte, and Platelet (HALP) score has emerged as a useful prognostic biomarker in metastatic lung cancer, with low baseline levels linked to poor outcomes, dynamic increases during treatment associated with superior survival, and consistent prognostic value in metastatic squamous cell carcinoma. However, its predictive utility appears limited in small cell lung cancer, where metastatic sites such as liver and brain metastases more strongly influence clinical outcomes (16, 17). Furthermore, Aberrant coagulation function also contributes to tumor progression and metastasis through mechanisms such as promoting angiogenesis, regulating growth factors, and inhibiting natural killer cell activity (18–20). Fibrinogen (FIB) and D-dimer (D-D) are key biomarkers of hypercoagulability and secondary fibrinolysis (21, 22). Fibrinogen facilitates immune evasion by tumor cells and supports angiogenesis, whereas D-dimer maintains a sustained hypercoagulable state that fosters a permissive microenvironment for metastatic dissemination (23, 24). Emerging evidence suggests that the D-dimer-to-fibrinogen ratio (DFR) may serve as a more sensitive indicator of coagulation–fibrinolysis imbalance and often outperforms single parameters in predicting metastasis (25). In addition, tumor-associated thrombocytosis is closely correlated with advanced disease stage, metastatic status, and poor prognosis (26). Conventional serum tumor markers—such as Carcinoembryonic Antigen (CEA), Squamous Cell Carcinoma Antigen (SCCA), Cytokeratin 19 fragment (CYFRA21-1), and Neuron-specific enolase (NSE)—are significantly correlated with tumor burden and metastatic spread in lung cancer (27–29). Importantly, the combined detection of multiple markers has been shown to enhance the sensitivity and specificity of diagnostic and monitoring strategies (27, 29, 30).

Although the aforementioned tumor-related individual indicators have demonstrated diagnostic potential for identifying baseline metastasis, existing studies predominantly focus on efficacy evaluation or prognostic analysis in advanced lung cancer, leaving a notable gap in their application for pre-treatment risk stratification at the time of initial diagnosis. Several investigators have applied radiomics or genomic features integrated with ML algorithms to achieve favorable performance in predicting intrapulmonary metastasis of lung adenocarcinoma or lymph node metastasis in non-small cell lung cancer (AUC > 0.81), and have further elucidated metastasis-related imaging phenotypes, gene mutations, and metabolic pathway alterations (31–33). Nevertheless, these approaches predominantly depend on imaging or invasive tissue/liquid biopsy techniques, which are constrained by high costs, radiation exposure, limited sampling frequency, or substantial technical barriers, thereby hindering their widespread implementation for early metastasis risk warning. Accurate baseline identification of distant metastasis is of paramount clinical importance in lung cancer management, as the presence of metastatic disease at initial diagnosis dictates the shift from curative-intent local therapy to systemic palliative treatment; however, current standard imaging-based staging has inherent limitations in detecting occult micrometastases, and advanced modalities such as PET-CT, though more sensitive, are often restricted by high costs and limited accessibility. Therefore, a cost-effective, non-invasive triage tool capable of stratifying patients according to baseline metastasis risk could facilitate personalized staging workup and optimize resource allocation. Yet, studies employing machine learning to construct combined models that integrate inflammatory, immunonutritional, coagulation-fibrinolysis, and tumor marker parameters for this purpose remain scarce. To address this gap, the present study developed and validated a diagnostic triage framework based on nine machine learning algorithms using clinically accessible, repeatable, and low-cost laboratory indicators, with the aim of identifying patients at high risk of occult metastasis who require intensified staging workup. Furthermore, the SHAP method was employed to identify the optimal set of key feature variables and to quantify both the magnitude and direction of each feature’s contribution to the model output (34).

## Materials and methods

### Clinical data

Patients were stratified into two groups according to the presence or absence of distant metastasis at initial diagnosis. Demographic characteristics (age, gender, height, weight, smoking history, drinking history), histological type, and pretreatment venous blood parameters were collected, including ALB, GLB, FIB, D-D, PLT, Neutrophil, Lymphocyte, Monocyte counts, and serum tumor markers (CEA, CYFRA21-1, NSE, and SCCA). Based on these indicators, a series of composite indices were calculated: SII, NLR, LMR, PLR, SIRI, AGR, PNI, HALP and BMI.

### Diagnostic criteria

Inclusion criteria were defined as follows: (1) patients with a histologically or cytologically confirmed diagnosis of primary bronchogenic carcinoma, established via fiberoptic bronchoscopy, percutaneous lung biopsy, surgical biopsy, sputum exfoliative cytology, or pleural effusion cytology; (2) availability of complete clinical records and pretreatment imaging and laboratory assessments, including mandatory contrast-enhanced computed tomography (CT) of the chest and whole abdomen, and contrast-enhanced magnetic resonance imaging (MRI) or CT of the brain, all performed within one week prior to treatment initiation; (3) additional staging examinations—including whole-body bone scintigraphy, PET-CT, pleural biopsy, thoracoscopy, or pleural effusion cytology—were performed selectively based on clinical indications to confirm or rule out suspected metastatic lesions. (4) all included patients had complete and evaluable metastatic assessment data, with no diagnostic ambiguity regarding the presence or absence of distant metastases.

Exclusion criteria were defined as follows: (1) prior receipt of chemotherapy, radiotherapy, or surgical resection; (2) presence of concurrent hematological disorders, liver diseases, or infectious diseases; (3) administration of albumin or globulin infusion within the preceding 3 months; (4) diagnosis of recurrent or metastatic lung cancer; (5) history of other primary malignancies; (6) history of myocardial infarction, arterial thromboembolism, or venous thromboembolism within 3 months prior to treatment initiation; (7) current use of anticoagulant or antiplatelet therapy; (8) incomplete or unavailable clinical data, or metastatic lesion assessment that did not meet the standardized diagnostic criteria described; (9) equivocal metastatic findings without definitive histological confirmation, or without concordant findings from at least two imaging modalities.

### Definition of distant metastasis

According to the 8th edition of the TNM, distant metastasis was defined as the presence of metastatic lesions in the brain, bone, liver, adrenal glands, pleura, or other distant organs. The diagnosis of metastasis was established using the following standardized criteria:

1. Definitive metastasis: histological confirmation via biopsy or surgical specimen of the suspected lesion.
2. Suspicious imaging findings without histological confirmation: at least two different imaging modalities were required to concurrently suggest metastasis.

All metastatic cases included in the analysis were supported by adequate histological or imaging evidence according to the above criteria, thereby eliminating the risk of misclassification due to diagnostic uncertainty. The diagnostic modalities used for detection included contrast-enhanced CT or MRI of the brain, contrast-enhanced CT of the chest and abdomen, whole-body bone scintigraphy, PET-CT, pleural biopsy, thoracoscopy, or pleural effusion cytology.

### Variable screening and model development

**Figure 1:**
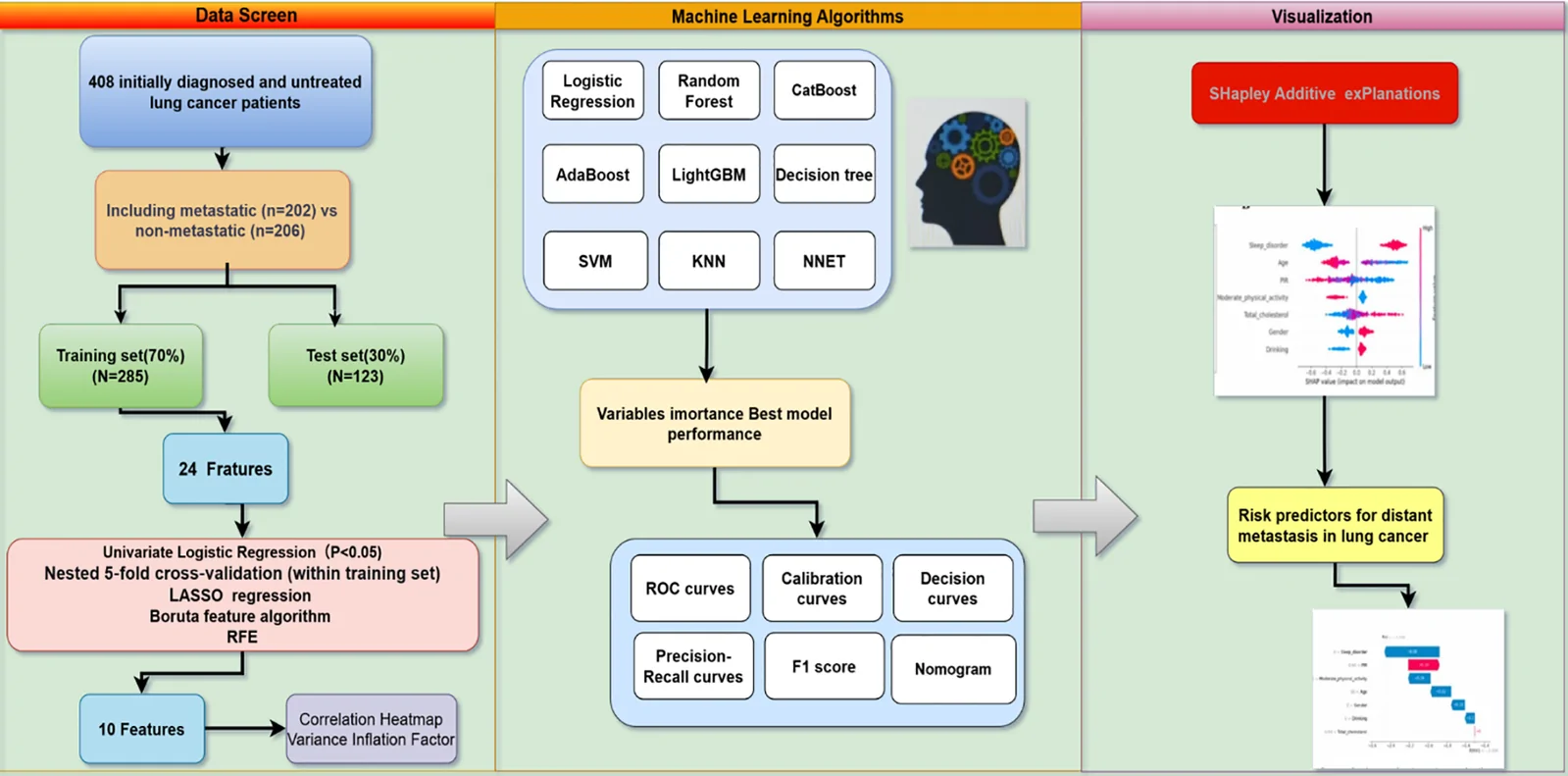
The overall flowchart of the study.

The data preprocessing and modeling workflow is presented in **Figure 1**. All patient records were obtained from the hospital’s electronic medical record system. During the initial data quality control phase, we predefined an exclusion criterion that any variable with more than 20% missing values would be removed. However, in the final analytical dataset of 408 patients, all 24 candidate variables had no missing data; therefore, no variables were excluded and no imputation was performed.

A total of 408 lung cancer patients were enrolled, including 206 without distant metastasis and 202 with distant metastasis. Patients were randomly assigned to a training set (n = 285, 70%) and an independent test set (n = 123, 30%) using stratified random sampling to preserve the distribution of the outcome variable. The test set was strictly isolated from the outset and was never involved in any feature selection, model development, or algorithm comparison, being reserved exclusively for the final single-run performance evaluation of the locked model.

To avoid information leakage and obtain unbiased performance estimates, we implemented a fully nested 5-fold cross-validation strategy within the training set. This approach served two purposes: first, to perform feature selection independently within each fold to ensure that the validation set information did not contaminate the screening process; and second, to compare the nine candidate machine learning algorithms and identify the optimal classifier. Within each fold, we first used only the training partition of that fold to independently execute a three-pronged feature selection pipeline, which included univariate logistic regression retaining variables with P < 0.05, LASSO regression, the Boruta algorithm, and recursive feature elimination, retaining only those features concurrently selected by all three methods. Based on the selected features, nine algorithms—Decision Tree, K-Nearest Neighbors, LightGBM, Logistic Regression, Random Forest, Support Vector Machine, Neural Network, AdaBoost, and CatBoost—were then trained on the same training partition, with hyperparameter tuning performed via grid search combined with inner 3-fold cross-validation. Finally, the tuned models were evaluated on the corresponding validation partition and the AUC was recorded. This procedure was repeated for each of the five folds, yielding five validation AUCs for each algorithm. The mean AUC across the five validation folds served as the internal validation metric for algorithm comparison, and the algorithm with the highest mean value was selected as the optimal classifier.

After identifying the optimal algorithm and its predictor set based on the nested CV results, we retrained the final model on the full training set. The locked model was then evaluated once on the independent test set, and this test-set AUC was reported as the final, unbiased estimate of generalizability. Model performance was assessed using the area under the ROC curve with its 95% confidence interval, accuracy, F1 score, sensitivity, specificity, and Youden index. On the independent test set, the DeLong test was employed to compare AUC differences between models.

To facilitate bedside clinical application, we constructed a nomogram based on the final AdaBoost model, providing an intuitive scoring system for individualized risk estimation. Since AdaBoost is a nonlinear ensemble algorithm that does not directly produce interpretable regression coefficients, we employed a surrogate model approach to derive the nomogram. Specifically, we first applied the trained AdaBoost model to the entire training set to generate predicted probabilities of baseline distant metastasis for each patient. Subsequently, using the 10 features as independent variables and the logit of the predicted probabilities from the AdaBoost model as the dependent variable, we fitted a logistic regression model to obtain surrogate regression coefficients for each predictor. Finally, the nomogram was constructed based on these surrogate coefficients, with each predictor assigned a point value proportionally mapped from its regression coefficient; the sum of points across all predictors corresponded to the predicted probability of distant metastasis. This approach preserves the predictive performance of the nonlinear AdaBoost model while enabling clinical interpretability through a familiar nomogram format.

### Statistical analysis

All statistical analyses were performed using R software (version 4.4.1) for machine learning modeling and IBM SPSS Statistics (version 26.0) for baseline descriptive statistics and group comparisons; SPSS was not involved in any modeling steps. The Shapiro–Wilk test was employed to assess the normality of continuous variables. Normally distributed variables are expressed as mean ± standard deviation (SD), while non-normally distributed variables are expressed as median and interquartile range (IQR, 25th–75th percentile). Categorical variables are summarized as frequencies and percentages. Group comparisons were made using the chi-square test or Fisher’s exact test for categorical data, the independent samples t-test for normally distributed continuous data, and the Mann–Whitney U test for non-normally distributed continuous data.

For machine learning model development, a unified random seed [set.seed(2024)] was applied across all stochastic steps to ensure full reproducibility. The complete dataset was randomly split into a training set and a test set using stratified random sampling to preserve the distribution of the outcome variable. All 24 candidate variables had 0% missingness; therefore, no imputation methods were applied. Scale-sensitive algorithms (SVM and KNN) underwent standardization (centered and scaled to unit variance), while tree-based and ensemble methods were not scaled. No logarithmic or other transformations were applied, as the final recommended model is naturally robust to skewed distributions, and applying such transformations would have compromised clinical interpretability.

Hyperparameter tuning was performed using grid search combined with 5-fold stratified cross-validation, with cross-validated AUC maximization as the selection criterion. The final parameter configurations for all nine algorithms—including hyperparameter search spaces and optimal values—have been fully reported in **Supplementary Appendix S1**. The classification threshold was determined by maximizing the Youden index, with 0.65 recommended as the default reference threshold. No independent probability calibration step was performed, as the model already demonstrated satisfactory calibration in the test set (Hosmer–Lemeshow test P = 0.122, MAE = 0.057), and calibration curves and decision curve analysis were based on raw predicted probabilities.

## Result

### Comparison of baseline characteristics and laboratory parameters

A total of 408 patients with lung cancer were enrolled in this study (**Table 1**). Comparative analysis between the metastatic group (n = 202) and the non-metastatic group (n = 206) revealed that patients with distant metastasis had a significantly higher proportion of smoking history (64.8% vs. 35.2%, P < 0.001) and exhibited distinct distributions of histological types (P = 0.001). Moreover, levels of coagulation markers—including D-dimer, fibrinogen, and platelet count—as well as tumor markers (CEA, CYFRA21-1, NSE) and systemic inflammation indices (SII, NLR, PLR, SIRI) were significantly elevated in the metastatic group (P < 0.001). Conversely, immunonutritional indicators such as LMR, ALB, AGR, PNI, and HALP were significantly decreased (P < 0.001). These findings suggest that patients with distant metastasis exhibit more pronounced systemic inflammation and hypercoagulability, poorer nutritional and immune status, and a higher tumor burden. Following imbalance processing, all cases were randomly assigned to a training set (n = 285) and a test set (n = 123) at a 7:3 ratio, with no significant differences in baseline characteristics observed between the two sets (**Table 2**).

**Table 1:** Baseline characteristics and laboratory parameters of lung cancer patients stratified by distant metastasis status [n (%) or median (IQR)].

Variables	Total (n = 408)	Non-metastatic (n = 206)	Metastatic (n = 202)	Statistic	P
ALB	38.17 ± 4.71	39.37 ± 4.89	36.94 ± 4.17	t=5.40	<.001
GLB	29.00 (26.40, 32.62)	28.45 (25.80, 32.18)	29.70 (26.80, 33.55)	Z=-2.75	0.006
AGR	1.30 (1.14, 1.50)	1.40 (1.20, 1.50)	1.27 (1.10, 1.40)	Z=-5.54	<.001
D-D	540.00 (250.00, 1242.50)	320.00 (220.00, 617.50)	880.00 (435.00, 2207.50)	Z=-8.43	<.001
FIB	3.91 (3.17, 4.82)	3.51 (2.97, 4.35)	4.42 (3.58, 5.20)	Z=-6.57	<.001
DFR	126.22 (78.69, 282.96)	98.37 (70.34, 161.55)	196.38 (103.31, 486.38)	Z=-6.82	<.001
PLT	261.00 (212.00, 319.00)	244.00 (192.00, 305.00)	273.50 (225.00, 326.75)	Z=-3.94	<.001
CEA	4.46 (2.42, 20.03)	3.08 (1.74, 6.38)	9.54 (3.84, 66.65)	Z=-8.57	<.001
CYFRA21-1	4.25 (2.81, 7.46)	3.25 (2.18, 4.57)	5.87 (4.06, 12.10)	Z=-9.29	<.001
NSE	14.35 (11.20, 21.26)	12.40 (10.43, 15.97)	18.15 (13.30, 30.93)	Z=-7.79	<.001
SCCA	1.27 (0.88, 2.09)	1.34 (0.93, 1.95)	1.23 (0.84, 2.30)	Z=-0.33	0.738
SII	745.98 (504.62, 1100.64)	617.40 (429.73, 953.84)	850.75 (608.11, 1236.40)	Z=-5.57	<.001
NLR	2.86 (2.17, 3.91)	2.60 (1.97, 3.54)	3.18 (2.43, 4.16)	Z=-4.64	<.001
LMR	3.01 (2.17, 4.05)	3.35 (2.48, 4.44)	2.58 (1.98, 3.48)	Z=-5.33	<.001
PLR	162.88 (122.52, 215.40)	150.96 (108.22, 204.64)	179.21 (133.92, 246.92)	Z=-3.97	<.001
SIRI	1.56 (0.99, 2.35)	1.29 (0.85, 1.85)	1.93 (1.23, 2.77)	Z=-6.19	<.001
HALP	30.09 (20.45, 41.65)	33.91 (23.92, 47.57)	26.76 (17.96, 37.71)	Z=-4.53	<.001
PNI	69.28 (51.57, 88.94)	73.52 (57.08, 94.60)	65.66 (49.06, 81.16)	Z=-3.25	0.001
BMI	22.16 ± 3.54	22.33 ± 3.78	21.99 ± 3.29	t=0.96	0.336
Gender				χ²=2.86	0.091
Male	256 (62.75)	121 (58.74)	135 (66.83)		
Female	152 (37.25)	85 (41.26)	67 (33.17)		
Age				χ²=2.53	0.112
≤60	124 (30.39)	70 (33.98)	54 (26.73)		
>60	284 (69.61)	136 (66.02)	148 (73.27)		
Histological type				χ²=13.66	0.001
Adenocarcinoma	273 (66.91)	149 (72.33)	124 (61.39)		
Squamous carcinoma	68 (16.67)	37 (17.96)	31 (15.35)		
Small cell lung cancer	67 (16.42)	20 (9.71)	47 (23.27)		
Smoking history				χ²=49.19	<.001
NO	178 (43.63)	125 (60.68)	53 (26.24)		
YES	230 (56.37)	81 (39.32)	149 (73.76)		
Drinking history				χ²=0.64	0.423
NO	333 (81.62)	165 (80.10)	168 (83.17)		
YES	75 (18.38)	41 (19.90)	34 (16.83)		

**Table 2:** Baseline characteristics and laboratory parameters of the training and validation cohorts [n (%) or median (IQR)].

Variables	Total (n = 408)	Training Data(n = 285)	Validation Data(n = 123)	Statistic	P
ALB	38.17 ± 4.71	38.21 ± 4.35	38.09 ± 5.46	t=0.24	0.814
GLB	29.00 (26.40, 32.62)	28.90 (26.50, 32.70)	29.30 (26.15, 32.30)	Z=-0.29	0.774
AGR	1.30 (1.14, 1.50)	1.30 (1.10, 1.50)	1.32 (1.17, 1.50)	Z=-0.47	0.642
D-D	540.00 (250.00, 1242.50)	530.00 (230.00, 1230.00)	560.00 (295.00, 1410.00)	Z=-1.03	0.303
FIB	3.91 (3.17, 4.82)	3.92 (3.17, 4.83)	3.91 (3.21, 4.76)	Z=-0.01	0.994
DFR	126.22 (78.69, 282.96)	120.48 (78.57, 280.00)	134.02 (79.03, 313.80)	Z=-0.77	0.439
PLA	261.00 (212.00, 319.00)	260.00 (212.00, 317.00)	266.00 (213.50, 325.50)	Z=-0.36	0.719
CEA	4.46 (2.42, 20.03)	4.44 (2.42, 20.70)	4.77 (2.42, 17.45)	Z=-0.34	0.734
CYFRA21-1	4.25 (2.81, 7.46)	4.32 (2.75, 7.52)	4.10 (2.88, 7.39)	Z=-0.24	0.808
NSE	14.35 (11.20, 21.26)	14.70 (11.20, 21.25)	13.70 (11.25, 21.35)	Z=-0.48	0.631
SCCA	1.27 (0.88, 2.09)	1.24 (0.88, 2.08)	1.35 (0.92, 2.20)	Z=-0.97	0.333
SII	745.98 (504.62, 1100.64)	713.33 (515.38, 1084.60)	804.01 (480.94, 1181.48)	Z=-0.55	0.581
NLR	2.86 (2.17, 3.91)	2.82 (2.18, 3.80)	2.92 (2.12, 3.95)	Z=-0.46	0.648
LMR	3.01 (2.17, 4.05)	3.12 (2.21, 4.09)	2.85 (2.12, 3.92)	Z=-1.37	0.17
PLR	162.88 (122.52, 215.40)	160.30 (122.57, 214.20)	168.07 (124.38, 220.89)	Z=-0.44	0.661
SIRI	1.56 (0.99, 2.35)	1.56 (0.97, 2.28)	1.53 (1.06, 2.59)	Z=-1.13	0.259
HALP	30.09 (20.45, 41.65)	30.79 (20.68, 41.22)	29.01 (19.61, 44.75)	Z=-0.36	0.721
PNI	69.28 (51.57, 88.94)	69.16 (51.50, 90.64)	69.39 (52.16, 88.38)	Z=-0.27	0.784
BMI	22.16 ± 3.54	22.10 ± 3.35	22.30 ± 3.97	t=-0.50	0.616
Gender				χ²=0.73	0.394
Male	256 (62.75)	175 (61.40)	81 (65.85)		
Female	152 (37.25)	110 (38.60)	42 (34.15)		
Age				χ²=1.17	0.279
≤60	284 (69.61)	203 (71.23)	81 (65.85)		
>60	124 (30.39)	82 (28.77)	42 (34.15)		
Histological type				χ²=0.39	0.825
Adenocarcinoma	273 (66.91)	188 (65.96)	85 (69.11)		
Squamous carcinoma	68 (16.67)	49 (17.19)	19 (15.45)		
Small cell lung cancer	67 (16.42)	48 (16.84)	19 (15.45)		
Smoking history				χ²=0.34	0.563
NO	178 (43.63)	127 (44.56)	51 (41.46)		
YES	230 (56.37)	158 (55.44)	72 (58.54)		
Drinking history				χ²=0.03	0.865
NO	333 (81.62)	232 (81.40)	101 (82.11)		
YES	75 (18.38)	53 (18.60)	22 (17.89)		

### Univariate logistic regression analysis

Univariate logistic regression analysis identified multiple variables significantly associated with distant metastasis in lung cancer (**Table 3**). Notable predictors included smoking history, histological type (particularly small cell lung cancer), and a range of laboratory parameters. Systemic inflammation indices—such as SIRI, NLR, and PLR—were significantly elevated in metastatic patients. Tumor markers, including CEA, CYFRA21-1 and NSE, also demonstrated strong associations with metastasis. Furthermore, coagulation markers (FIB and D-D) and immunonutritional indicators (ALB, AGR and PNI) emerged as significant predictors (P < 0.05). In contrast, demographic variables including Gender, Age, Drinking history, and BMI showed no statistically significant association with metastasis status. Variables with P < 0.05 were subsequently considered for further feature selection and multivariable modeling.

**Table 3:** Univariate logistic regression analysis for distant metastasis.

Variable	OR (95% CI)	Wald	P
Albumin (g/L)	0.884 (0.833–0.937)	−5.05	<0.001
Globulin (g/L)	1.083 (1.029–1.140)	2.98	0.003
Albumin-to-globulin ratio	0.085 (0.031–0.239)	−4.87	<0.001
D-dimer (μg/L)	1.001 (1.001–1.001)	4.13	<0.001
Fibrinogen (g/L)	1.739 (1.399–2.162)	5.70	<0.001
D-dimer-to-fibrinogen ratio	1.003 (1.001–1.004)	2.38	0.017
Platelet (×10^9^/L)	1.004 (1.002–1.006)	3.40	<0.001
CEA (ng/mL)	1.008 (1.002–1.014)	4.00	<0.001
CYFRA21-1 (ng/mL)	1.135 (1.070–1.203)	5.09	<0.001
NSE (ng/mL)	1.018 (1.006–1.030)	3.62	<0.001
SCCA (ng/mL)	1.024 (0.983–1.067)	0.96	0.339
Systemic immune-inflammation index	1.001 (1.001–1.001)	5.17	<0.001
Neutrophil-to-lymphocyte ratio	1.449 (1.208–1.739)	4.33	<0.001
Lymphocyte-to-monocyte ratio	0.677 (0.563–0.814)	−4.59	<0.001
Platelet-to-lymphocyte ratio	1.006 (1.002–1.010)	4.13	<0.001
Systemic inflammation response index	2.049 (1.548–2.711)	5.54	<0.001
HALP	0.972 (0.957–0.988)	−4.38	<0.001
Prognostic nutritional index	0.988 (0.978–0.998)	−3.18	0.001
BMI (kg/m²)	0.979 (0.914–1.048)	−0.96	0.335
Gender			
*Male*	Ref		
*Female*	0.628 (0.389–1.015)	−1.69	0.091
Age			
*≤ 60 years*	Ref		
> *60 years*	0.716 (0.428–1.199)	−1.59	0.112
Histological type			
*Adenocarcinoma*	Ref		
*Squamous carcinoma*	0.890 (0.471–1.679)	0.02	0.980
*Small cell lung cancer*	3.193 (1.589–6.416)	3.54	<0.001
Smoking history			
*No*	Ref		
*Yes*	7.314 (4.308–12.415)	6.85	<0.001
Drinking history			
*No*	Ref		
*Yes*	0.878 (0.483–1.596)	−0.80	0.424

### Feature selection

**Figure 2:**
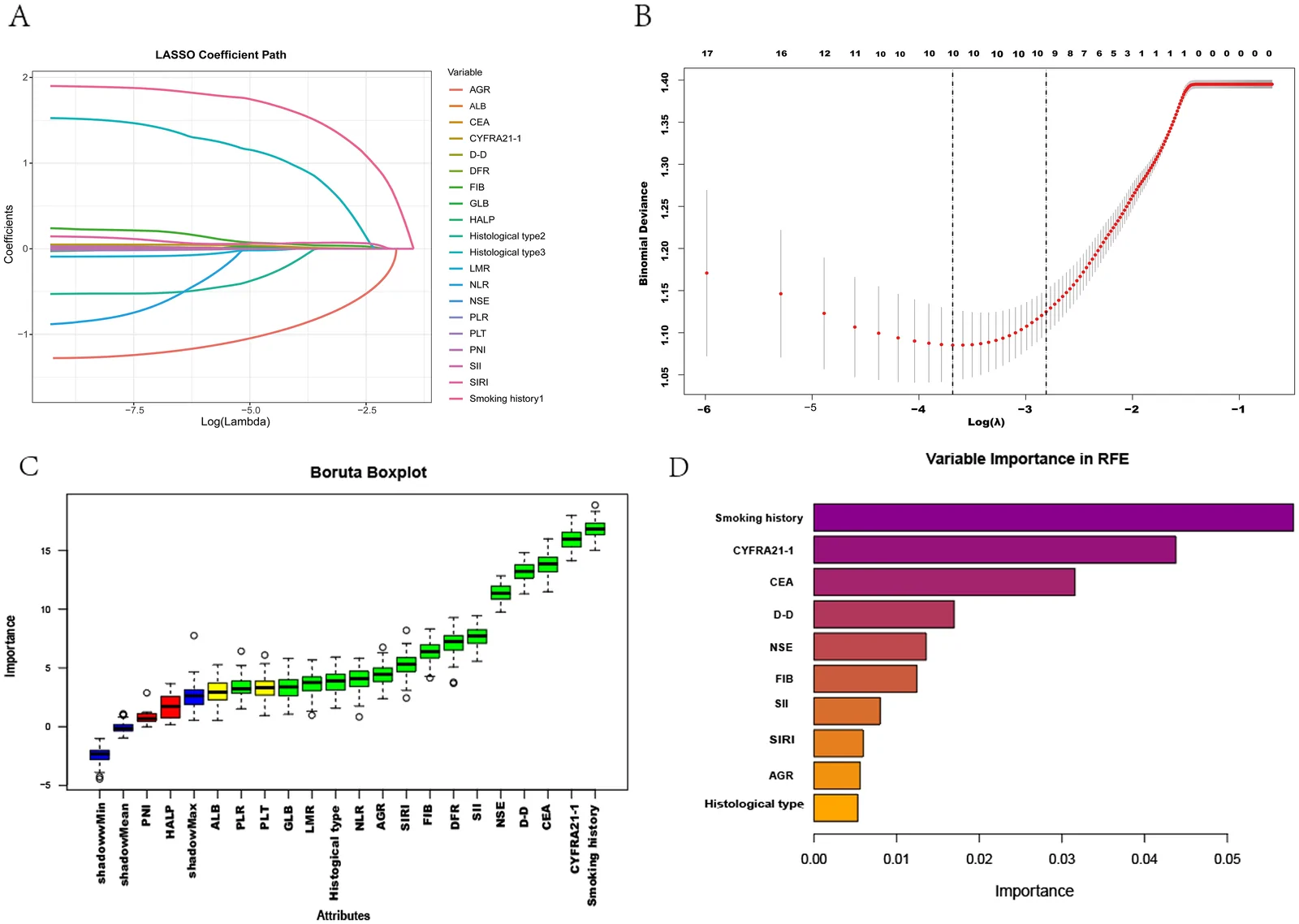
Feature selection process. **(A)** Coefficient profile plot of the LASSO regression model. **(B)** Cross-validation plot for tuning parameter (λ) selection in the LASSO model. The left dashed line indicates the optimal λ value (lambda.min), and the right dashed line represents the λ value within one standard error of the minimum (lambda.1se). **(C)** Boruta variable selection plot, showing the importance of confirmed, tentative, and rejected attributes. **(D)** Recursive feature elimination (RFE) variable selection plot, illustrating the change in model accuracy across different numbers of features.

**Figure 3:**
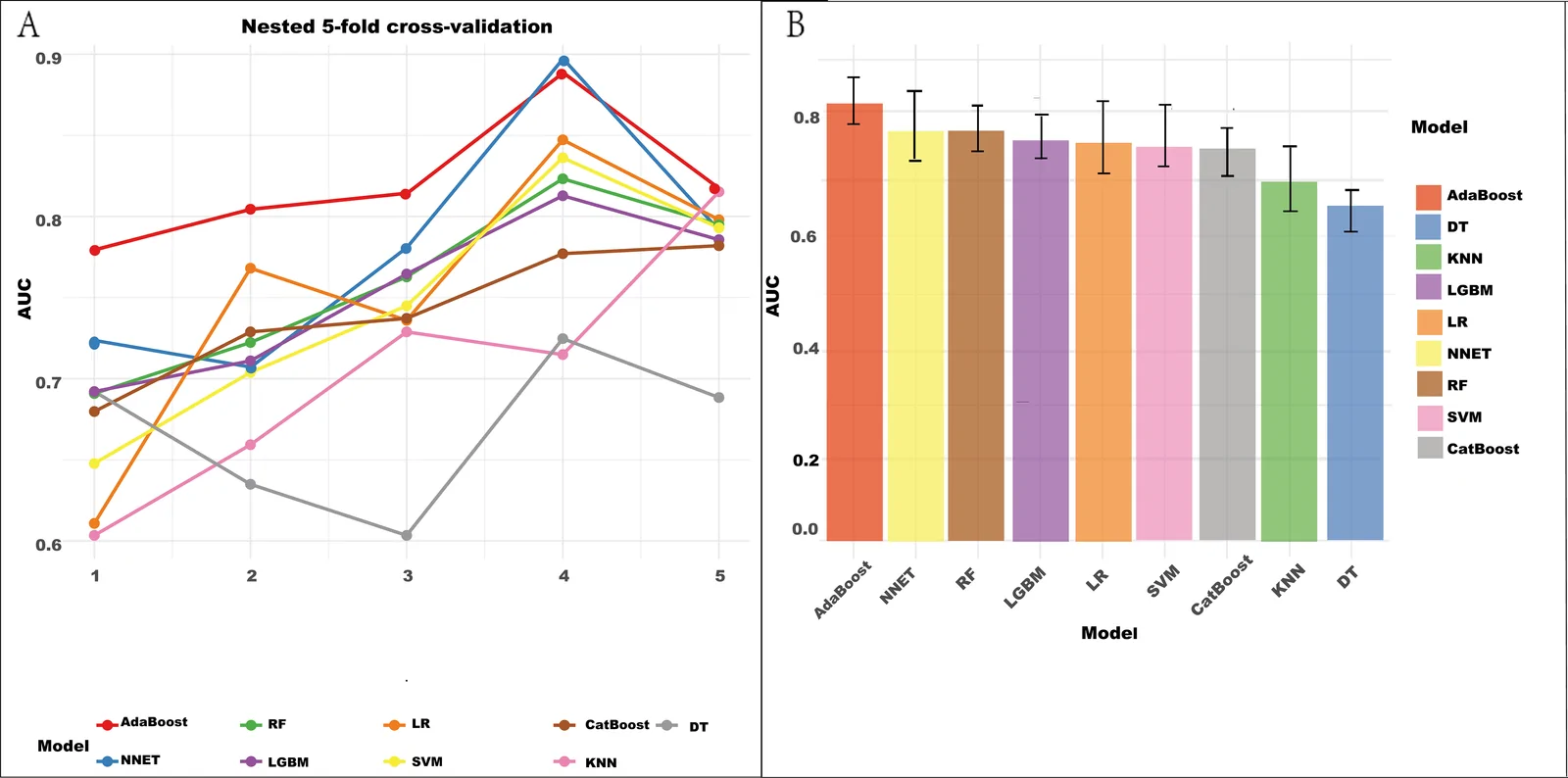
**(A)** Per-fold classification accuracy of nine classifier configurations across the five outer test folds of the nested 5-fold cross-validation. The inner loop of the nested scheme was dedicated to hyperparameter optimization via grid search. **(B)** Mean accuracy and fold-to-fold variability (error bars represent standard deviation, SD) of each classifier, aggregated from the five outer folds of the same nested cross-validation experiment shown in **(A)**.

Before the nested cross-validation procedure, we first performed univariate logistic regression screening on the entire training set to identify variables significantly associated with distant metastasis, retaining those with P < 0.05. Subsequently, within the nested 5-fold cross-validation framework, feature selection was performed independently in each fold using the retained variables, applying three complementary algorithms—LASSO regression, the Boruta algorithm, and recursive feature elimination—in parallel (**Figure 2**), with only features concurrently selected by all three methods retained for that fold. Across the five folds, AdaBoost consistently achieved the highest mean AUC (0.821, SD = 0.033, 95% CI: 0.744–0.828), outperforming all other candidate. The feature screening process consistently identified 10 key predictors: smoking history, histological type, D-dimer, fibrinogen, AGR, CEA, CYFRA21-1, NSE, SII, and SIRI (**Figure 3**).

### Evaluation of multicollinearity

**Figure 4:**
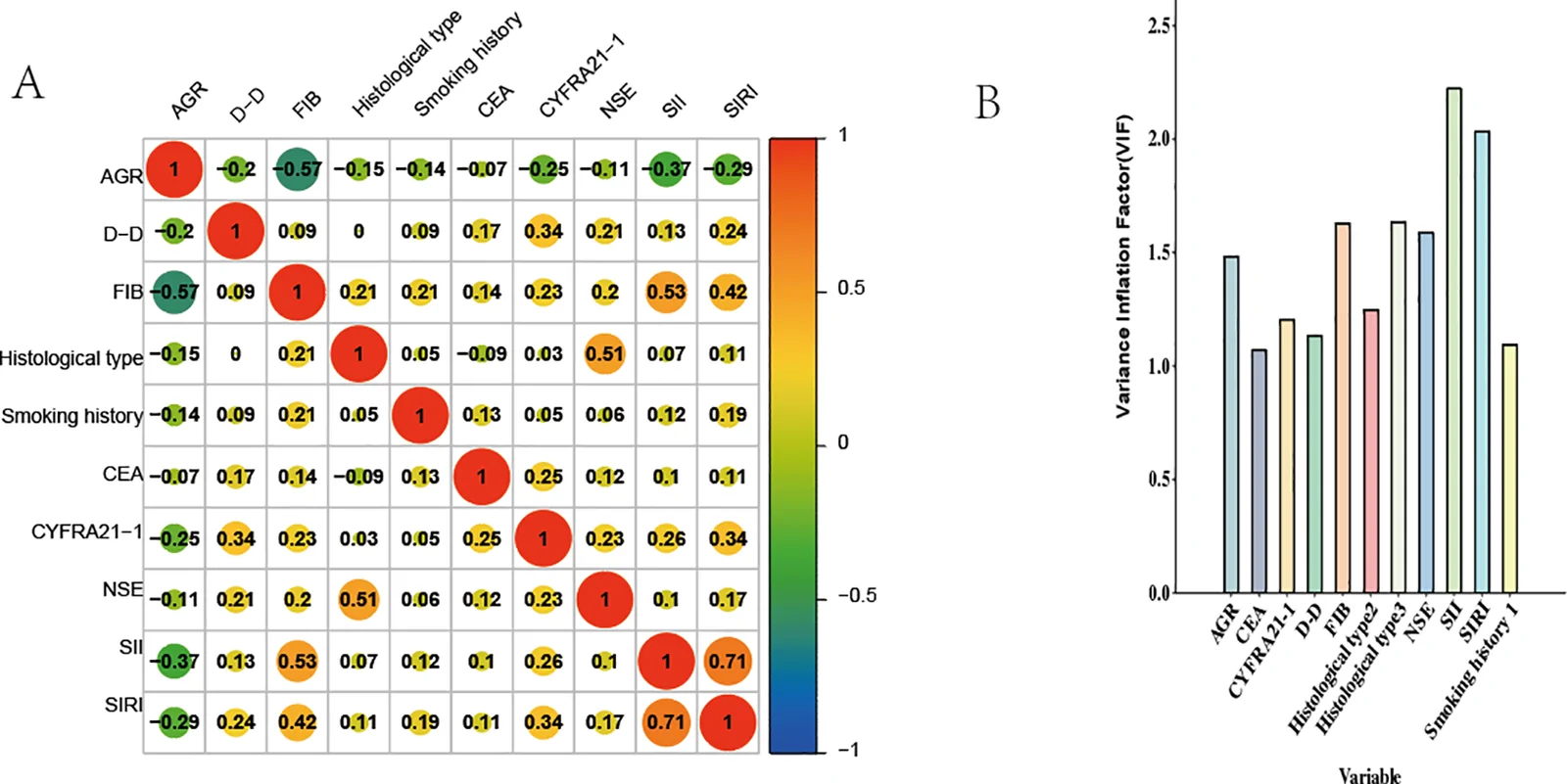
Correlation matrix and multicollinearity assessment of the selected predictors. **(A)** Pearson correlation matrix of the 10 candidate features. Red and blue colors represent positive and negative correlations, respectively, with darker shades indicating stronger correlations. **(B)** Variance inflation factor (VIF) values for each predictor. All VIF values were below 2.5, indicating no severe multicollinearity among the selected features.

To ensure the stability of the subsequent modeling process, multicollinearity among the candidate predictors was rigorously examined. Pearson correlation analysis (**Figure 4A**) revealed several noteworthy associations, including a strong positive correlation between SII and SIRI, moderate positive correlations of both SII and SIRI with FIB, and predominantly negative correlations of AGR with other variables. To quantify these interdependencies, variance inflation factors (VIF) were calculated (**Figure 4B**). With all VIF values falling below 2.5, no significant multicollinearity was detected. Consequently, all 10 features were retained and entered into the final model.

### Performance comparison of machine learning models

**Figure 5:**
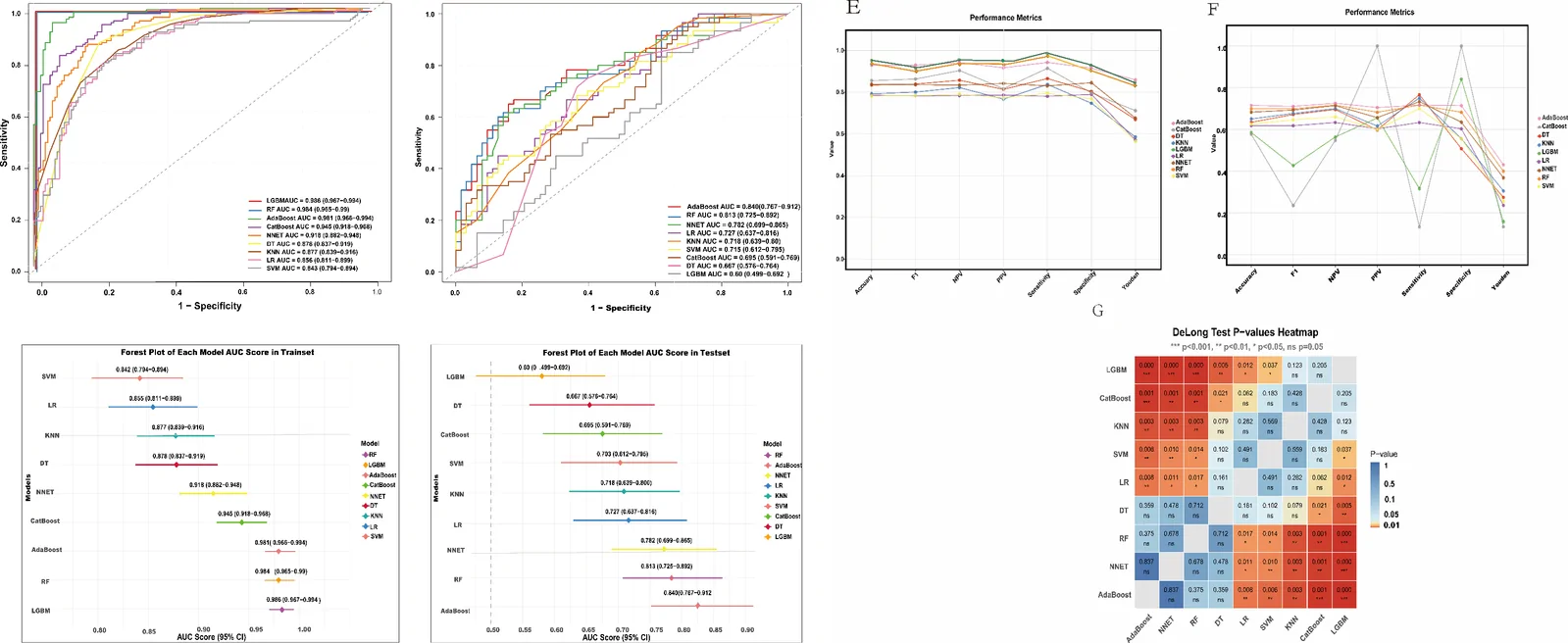
Performance comparison of nine machine learning models for baseline metastasis detection and risk triage. **(A)** ROC curves of the nine models on the training set. **(B)** ROC curves on the test set. **(C)** Forest plot of AUC values for the test set. **(D)** Forest plot of AUC values for the validation set. **(E)** Performance metrics (accuracy, F1 score, sensitivity, specificity, Youden index) of the nine models on the training set. **(F)** Performance metrics on the test set. **(G)** Heatmap of Delong’s test P-values for pairwise comparisons of model AUCs on the test set.

After algorithm selection was completed via nested 5-fold cross-validation, we retrained each model on the full training set and compared their performance on the independent test set to evaluate their generalizability in real-world clinical scenarios (**Table 4**; **Figure 5**). On the test set, AdaBoost achieved the best overall performance with an AUC of 0.840, accuracy of 0.715, sensitivity of 71.7%, specificity of 71.4%, and a Youden index of 0.431, demonstrating well-balanced metrics across all evaluation dimensions. Random Forest ranked second with an AUC of 0.813 and accuracy of 0.699. Neural Network (AUC = 0.782) and KNN (AUC = 0.718) also showed moderate generalizability. In contrast, Logistic Regression (AUC = 0.716), SVM (AUC = 0.715), and Decision Tree (AUC = 0.667) exhibited relatively limited discriminative ability. LightGBM showed a substantial drop in test AUC to 0.600, with a training-test gap of 0.386, suggesting severe overfitting. CatBoost, despite a specificity of 97.0%, achieved a sensitivity of only 13.3%, indicating severe prediction imbalance; neither model was suitable for clinical triage application. **DeLong pairwise tests further confirmed that AdaBoost significantly outperformed CatBoost (p = 0.001), KNN (p = 0.003), LR (p = 0.008), SVM (p = 0.008), and LGBM (p = 0.0001), while its differences from RF (p = 0.375), NN (p = 0.837), and DT (p = 0.359) were not statistically significant (**Table 5**). ** Notably, although AdaBoost showed a training-test AUC gap of 0.141, its test AUC of 0.840 remained clinically meaningful and consistently outperformed all other models. Given the modest sample size (n = 408), this gap is a common manifestation of the inherent complexity of ensemble algorithms rather than a specific indicator of overfitting. More importantly, the nested cross-validation results (mean AUC = 0.821) closely aligned with the independent test result (0.840), further supporting the reliability of the model’s generalizability. Among all algorithms, AdaBoost achieved the best trade-off between training accuracy and test generalizability, supporting its selection as the final model for subsequent evaluation. ,

**Table 4:** Model performance comparison between training and test sets.

Model	Set	AUC	Sensitivity	Specificity	Accuracy	PPV	NPV	F1	Youden
DT	*Training*	0.878	0.866	0.804	0.835	0.815	0.858	0.840	0.670
KNN	*Training*	0.877	0.838	0.748	0.793	0.768	0.823	0.801	0.586
LGBM	*Training*	0.986	0.974	0.916	0.950	0.928	0.932	0.921	0.850
LR	*Training*	0.856	0.782	0.790	0.786	0.787	0.785	0.784	0.572
RF	*Training*	0.984	0.969	0.910	0.940	0.920	0.920	0.910	0.840
SVM	*Training*	0.843	0.796	0.769	0.782	0.774	0.791	0.785	0.565
NNET	*Training*	0.918	0.831	0.846	0.839	0.843	0.834	0.837	0.677
**AdaBoost**	*Training*	**0.981**	0.944	0.916	0.930	0.918	0.942	0.931	**0.860**
CatBoost	*Training*	0.945	0.915	0.797	0.856	0.818	0.905	0.864	0.713
DT	*Test*	0.667	0.767	0.508	0.634	0.597	0.696	0.672	0.275
KNN	*Test*	0.718	0.750	0.556	0.650	0.616	0.700	0.677	0.306
LGBM	*Test*	0.600	0.317	0.841	0.585	0.655	0.564	0.427	0.158
LR	*Test*	0.716	0.633	0.603	0.618	0.603	0.633	0.618	0.237
RF	*Test*	0.813	0.717	0.683	0.699	0.683	0.717	0.699	0.399
SVM	*Test*	0.715	0.700	0.556	0.626	0.600	0.660	0.646	0.256
NNET	*Test*	0.782	0.733	0.635	0.683	0.657	0.714	0.693	0.368
**AdaBoost**	*Test*	**0.840**	0.717	0.714	0.715	0.705	0.726	0.711	**0.431**
CatBoost	*Test*	0.695	0.133	0.970	0.577	1.000	0.548	0.235	0.133

Best performance in the test set is shown in bold (AdaBoost: AUC = 0.840, Youden = 0.431). AdaBoost achieved well−balanced sensitivity (0.717) and specificity (0.714) in the test set,confirming its optimal generalization. CatBoost exhibited severe imbalance with very low sensitivity (0.133) despite high specificity (0.970), rendering it unsuitable for clinical application.

**Table 5:** Pairwise DeLong test results.

Independent test set • n = 123 • Bootstrap 95% CI(1000 resamples)				
Model	AUC	AUC diff. (vs. AdaBoost)	95% CI of diff.	*p*-value
AdaBoost	0.840 (0.770–0.910)	—	—	—
RF	0.813	0.027	−0.041 – 0.095	0.375
NNET	0.782	0.058	−0.014 – 0.130	0.837
CatBoost	0.741	0.099	0.035 – 0.163	0.001
KNN	0.718	0.122	0.046 – 0.198	0.003
LR	0.716	0.124	0.057 – 0.191	0.008
SVM	0.715	0.125	0.058 – 0.192	0.008
DT	0.667	0.173	0.089 – 0.257	0.359
LGBM	0.600	0.240	0.158 – 0.322	0.0001

AUC difference = AUC_comparator_ − AUC_AdaBoost_; positive values indicate AdaBoost outperforms the comparator. 95% CIs were estimated via bootstrap (1,000 resamples). *p*-values are raw (uncorrected) from the DeLong test.

### Performance of the AdaBoost model

**Figure 6:**
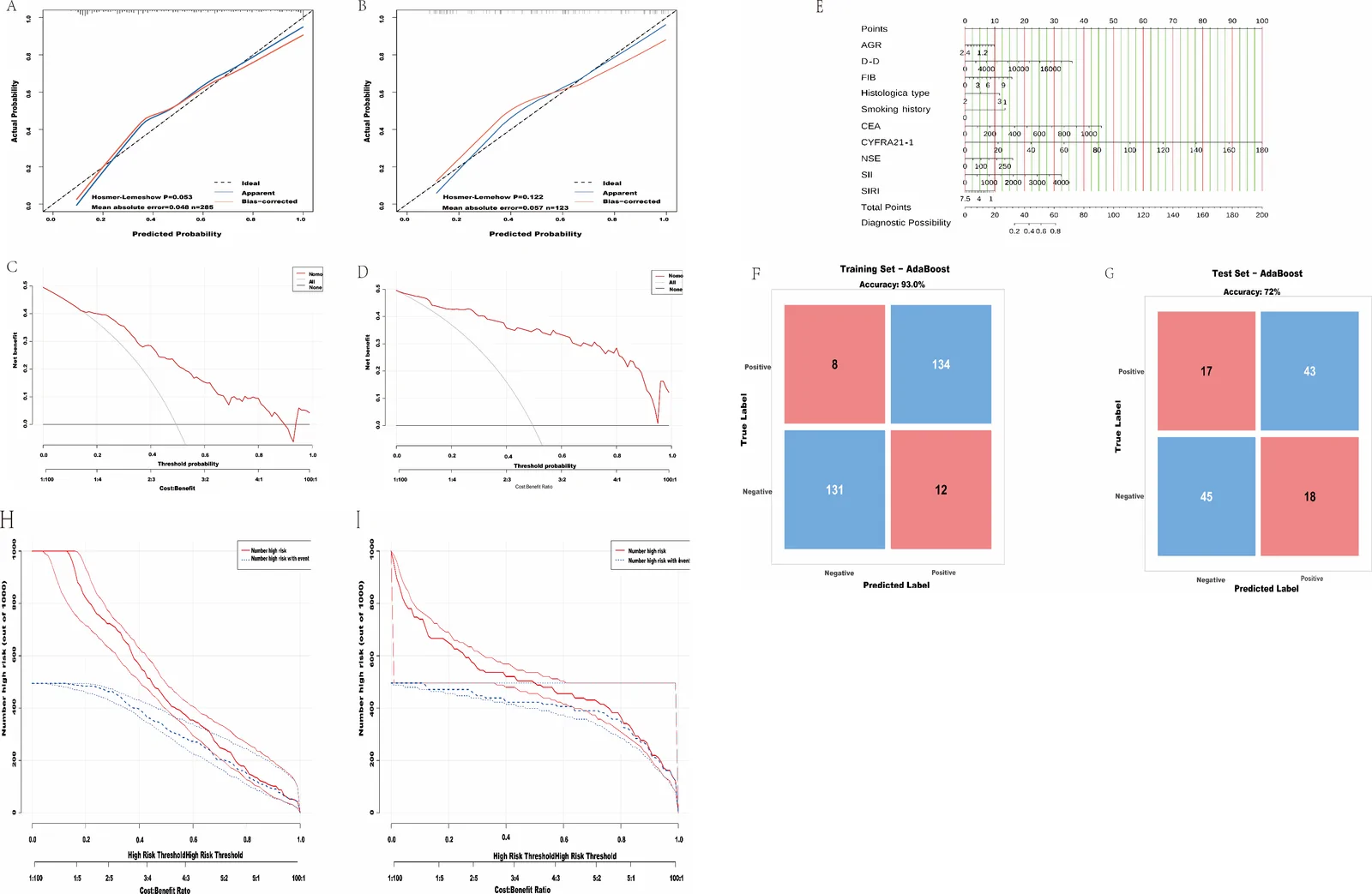
Performance evaluation of the AdaBoost model. **(A, B)** Calibration curves for the training and test sets, with apparent (solid line) and bias-corrected (dashed line) calibration estimated via bootstrap resampling; the diagonal dashed line indicates perfect prediction. **(C, D)** Decision curve analysis for the training and test sets, plotting net benefit (y-axis) against threshold probability (x-axis) to assess clinical utility. **(E)** Nomogram constructed based on the final AdaBoost model for individualized baseline distant metastasis risk assessment; each predictor variable is assigned a corresponding point value (upper scale), and the total points are summed to estimate the probability of distant metastasis (lower scale). **(F, G)** Confusion matrix analysis for the training and test sets, showing sensitivity and specificity values. **(H, I)** Clinical impact curves for the training and test sets, illustrating the number of individuals classified as high risk across threshold probabilities and the corresponding true positive counts.

The AdaBoost model demonstrated robust predictive performance and favorable clinical utility. Calibration curves for both the training and test sets revealed remarkable concordance between predicted probabilities and observed outcomes (**Figures 6A, B**), with Hosmer–Lemeshow test P-values of 0.053 and 0.122 and mean absolute errors of 0.048 and 0.057, respectively, confirming well-calibrated performance. Decision curve analysis showed that the model yielded substantial net clinical benefit across threshold probability ranges of 0.12–0.90 in the training set (**Figure 6C**) and 0.13–0.95 in the test set (**Figure 6D**). To enable intuitive bedside risk stratification, we constructed a nomogram based on the final AdaBoost model (**Figure 6E****),** which translates the algorithmic prediction into a visually interpretable scoring system: each predictor is assigned a weighted point value according to its regression coefficient, and the total point sum corresponds to the estimated probability of baseline distant metastasis, allowing clinicians to rapidly categorize patients into distinct risk tiers. Confusion matrix evaluation demonstrated that the model achieved a sensitivity of 94% and specificity of 82% in the training set (**Figure 6F**), while maintaining balanced performance in the test set, with sensitivity and specificity of 72% and 71%, respectively (**Figure 6G**). Clinical impact curves further corroborated the model’s clinical value, showing that at threshold probabilities above 60%, the number of high-risk individuals identified by the model closely approximated the actual number of metastasis cases (**Figures 6H, I****).**

### Feature importance and model interpretability

**Figure 7:**
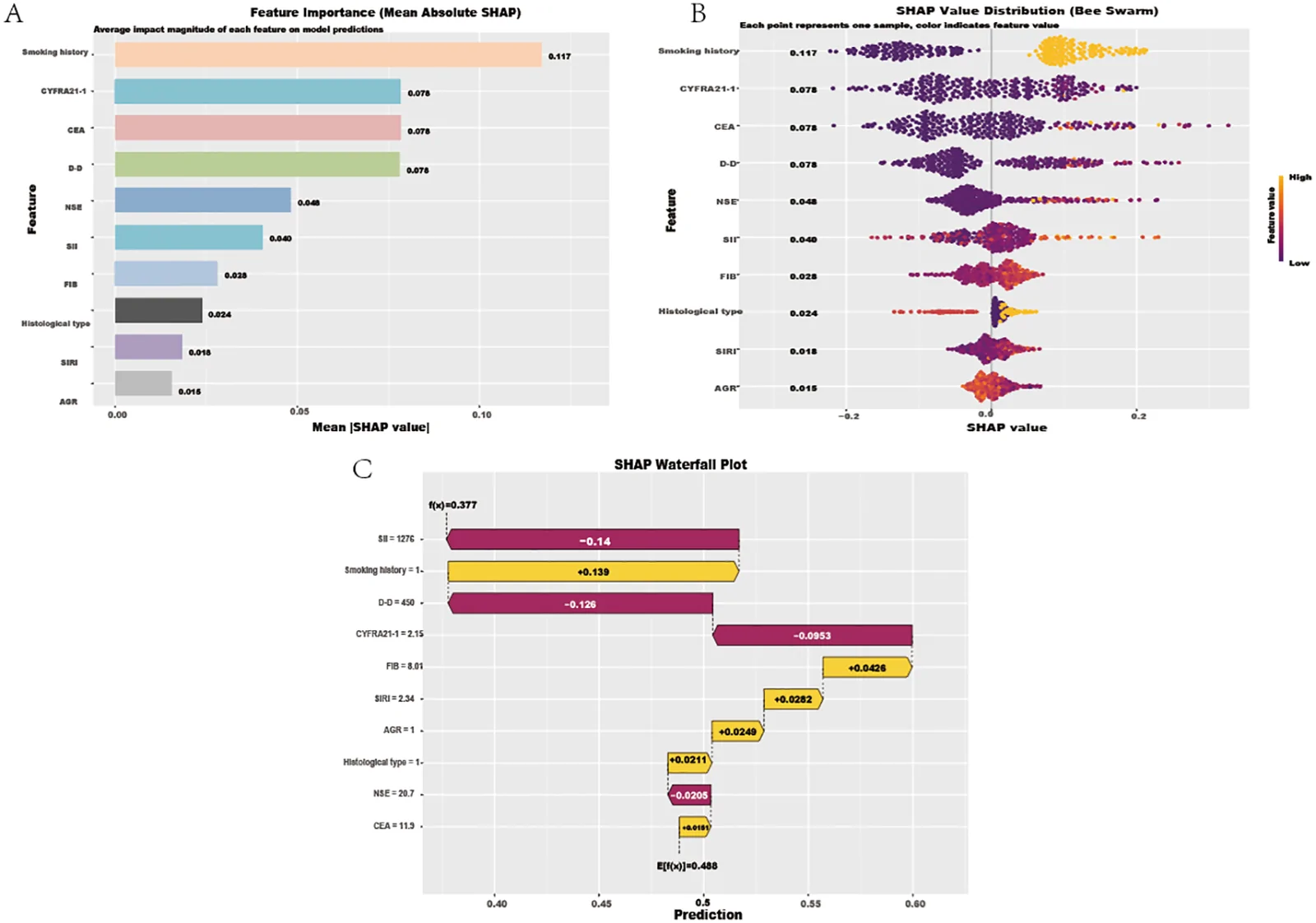
Feature interpretation in AdaBoost models. **(A)** Ranking of variable importance in AdaBoost. **(B)** Magnitude and direction of each variable’s influence on model output. **(C)** C.Examples of case interpretability analysis.

Feature importance was first evaluated using the AdaBoost algorithm, which identified smoking history, CEA, CYFRA21-1, D-dimer, and NSE as the five most influential predictors for identifying baseline metastasis (**Figure 7A**). To further elucidate the decision-making process of the optimal model, SHAP analysis was performed. The resulting feature importance ranking closely corroborated that obtained from AdaBoost (**Figure 7B****).** In the SHAP summary plot, each point represents the SHAP value for a given feature in an individual patient. The horizontal position reflects the magnitude and direction of the feature’s contribution to the model’s risk stratification output—points farther from the center exert greater influence. Color indicates the original feature value (yellow: high; purple: low), and the direction of clustering denotes the nature of the association captured by the model: rightward clustering signifies a positive contribution to the estimated metastasis risk, whereas leftward clustering indicates a negative contribution (i.e., lowering the risk estimate). For instance, smoking history (coded as 1) predominantly clustered to the right, reflecting a strong positive contribution to the model’s identification of baseline metastasis. Collectively, these results demonstrate that smoking history, along with elevated levels of CEA, CYFRA21-1, D-dimer, and NSE, exert the largest positive contributions to the model’s predictions, with smoking history exhibiting the highest predictive influence.

Individual-level interpretation was further illustrated using a SHAP waterfall plot (**Figure 7C****).** The baseline estimated probability of metastasis for the cohort was 0.377. For the representative sample shown, feature contributions are displayed as bars extending from this baseline; yellow bars represent positive contributions that increase the estimated risk, while purple bars denote negative contributions that decrease it. A high CEA level contributed +0.187 to the risk estimate, substantially elevating the risk assessment. Conversely, SII contributed –0.147, partially offsetting this increase. The cumulative effect of all feature contributions yielded a final estimated probability of 0.688 for this patient, indicating a markedly elevated baseline metastasis risk. The features ranked by contribution magnitude for this individual were: CEA > SII > smoking history > CYFRA21-1 > D-D > FIB > AGR > Histological type (adenocarcinoma) > SIRI > NSE, visually demonstrating the individualized impact of each variable on the model’s risk stratification output. It is important to note that SHAP is a model interpretation tool that quantifies the marginal contribution of each feature to the prediction output, rather than estimating causal effects between variables and outcomes.

## Discussion

Despite recent advances in radiotherapy and chemotherapy that have improved survival outcomes for patients with lung cancer, a substantial proportion of individuals present with distant metastasis at the time of diagnosis (35, 36). This is largely attributable to the insidious onset and nonspecific early symptoms of the disease, which collectively contribute to delayed detection, limited treatment efficacy, and poor prognosis. Distant metastasis in lung cancer is a complex, multistep biological cascade encompassing local tumor invasion, intravascular survival, immune evasion, and eventual colonization of distant organ microenvironments (37).

The initiation, progression, and metastasis of malignant tumors are closely intertwined with systemic inflammatory responses (38). Neutrophils contribute to tumor progression by suppressing immune activity, secreting pro-inflammatory cytokines, and facilitating the adhesion of circulating tumor cells; Lymphocytes serve as essential mediators of immune surveillance, and their depletion compromises anti-tumor immunity; Monocyte-derived macrophages secrete multiple tumor-promoting factors, while platelets protect tumor cells from mechanical and immune clearance and induce EMT; Collectively, these cellular components orchestrate a permissive microenvironment that drives distant metastasis (39–42).

The systemic SII and SIRI integrate key cell populations, quantifying the pro-inflammatory or immunosuppressive imbalance (4, 43). Elevated levels indicate neutrophilia/monocytosis with lymphopenia, promoting tumor invasion and metastasis via EMT, immune evasion, and colonization. Accumulating evidence has substantiated the prognostic value of these indices (44, 45). Wang et al (46)reported higher SII in extensive-stage SCLC than in limited-stage. Other studies showed SII independently predicts histological response to neoadjuvant immunotherapy in NSCLC, with low SII linked to major remission (47, 48). Pretreatment SIRI was identified as an independent risk factor for postoperative recurrence in early-stage lung cancer and for shorter PFS in advanced patients on targeted or immunotherapy (49). Notably, most evidence focuses on post-treatment prognosis. In contrast, our study confirms the association of elevated SII/SIRI with baseline distant metastasis and, for the first time, incorporates them into a pretreatment predictive risk (44, 50). This extends their utility to pre-treatment risk stratification, offering a novel tool for early high-risk identification.

Currently, evidence on the association between coagulation indicators and distant metastasis in lung cancer remains sparse. In our study, both D-D and FIB levels were significantly elevated in patients with distant metastasis (51). Coagulation abnormalities in advanced malignancies involve multiple mechanisms, including tumor-derived procoagulants, vascular injury, inflammatory cytokines, and circulating tumor cells (52). Elevated FIB promotes a hypercoagulable state, and its conversion to fibrin forms a network that traps tumor cells, facilitates immune evasion, and enhances metastatic colonization (23). Similarly, increased D-D reflects hyperfibrinolysis secondary to coagulation activation, and has been consistently linked to advanced stage, higher metastatic burden, and poor prognosis in lung cancer (51, 53). Shiina et al (54) demonstrated that elevated D-D was associated with vascular invasion and worse survival in NSCLC patients, even in stage IA disease. By integrating the mechanisms of D-D and FIB, our study elucidates their synergistic role in lung cancer metastasis (55). and provides a novel rationale for using routine coagulation-fibrinolysis parameters in early metastatic risk prediction.

Pretreatment malnutrition is widely recognized as a critical poor prognostic factor, affecting 21–45% of lung cancer patients (56, 57). Compared with albumin or globulin alone, the AGR offers a more integrated assessment by capturing patients with normal albumin but ongoing systemic inflammation and adverse prognosis. Substantial evidence links low AGR to tumor progression and poor outcomes across malignancies (14). For instance, a large cohort study by Zhou et al. (58) reported that lower AGR significantly correlated with shorter overall survival (derivation: HR = 1.35, 95% CI:1.01–1.81; validation: HR = 1.43, 95% CI:1.05–1.94) (58). Similarly, our study found significantly lower ALB and AGR in metastasis patients, with AGR as a protective factor (14, 58). An AGR cutoff of 1.29 has been validated for SCLC survival prediction, with HRs of 1.35 and 1.43 in two cohorts, remaining independent in multivariate analyses (58). Collectively, these findings suggest that the malnutrition and chronic inflammatory state reflected by a diminished AGR may promote tumor progression and metastatic dissemination through the impairment of host anti-tumor immunity (59). Thus, AGR is an accessible and valuable biomarker for metastasis risk stratification and individualized management.

Behavioral and intrinsic tumor characteristics are key predictors of distant metastasis in lung cancer (60, 61). Smoking history exhibited the largest positive SHAP contribution among all features, reflecting its dominant influence on the model’s identification of baseline metastasis. The biological pathways linking smoking to metastasis—including nicotine-driven phenotypic changes, angiogenesis, extracellular matrix degradation, and immune evasion—are well documented in previous studies (62).Furthermore, the incidence of distant metastasis varied significantly across histological subtypes. Adenocarcinoma and SCLC showed substantially higher rates than squamous cell carcinoma. This aligns with the known propensity of lung adenocarcinoma for hematogenous and pleural spread (63) and the aggressive, early-metastasizing nature of SCLC as a high-grade neuroendocrine tumor. Collectively, these findings highlight that intrinsic tumor biology fundamentally determines metastatic behavior (64, 65).

Accumulating evidence has demonstrated that the combined detection of multiple blood-based biomarkers significantly enhances the accuracy of identifying baseline distant metastasis in lung cancer. However, most existing studies have primarily focused on the prognostic value of these biomarkers after treatment initiation, leaving a critical gap in their application for pre-treatment risk stratification at the time of initial diagnosis (66). Previous studies have demonstrated that the combination of D-dimer and fibrinogen yields an AUC of 0.802 for identifying baseline metastasis in non-small cell lung cancer, substantially outperforming either marker alone (67, 68). Similarly, a multi-marker panel comprising CA125, CYFRA21-1, CEA, NSE, and ALP has been reported to achieve an AUC of 0.955 for identifying bone metastasis at initial diagnosis in lung cancer, with significantly improved sensitivity and specificity compared to individual markers (69). While these studies underscore the value of multi-marker strategies, existing models remain largely confined to predicting metastasis at a single anatomical site and rely on indicators drawn from a restricted range of biological dimensions. To address these limitations, the present study sought to develop a comprehensive diagnostic model that, for the first time, systematically integrates four distinct pathophysiological dimensions—systemic inflammation, tumor markers, coagulation function, and nutritional status—to identify baseline distant metastasis across multiple sites, thereby more fully capturing the systemic perturbations associated with metastatic dissemination. Our preliminary analyses confirmed that individual indicators exhibited only modest diagnostic performance (AUC range: 0.558–0.795), reinforcing the necessity of advanced integrative approaches. Accordingly, we employed machine learning techniques incorporating 24 candidate variables, encompassing demographic characteristics, histological type, and 19 serum biomarkers.

For feature selection, we first performed univariate logistic regression screening on the entire training set to identify variables significantly associated with distant metastasis, retaining those with P < 0.05 for further analysis. Subsequently, within the nested 5-fold cross-validation framework—in which feature selection and model training were conducted independently in each fold—we applied three complementary algorithms in parallel on the training partition of each fold: LASSO regression, the Boruta algorithm, and recursive feature elimination. Only features concurrently selected by all three methods were retained. This process consistently identified ten key predictors across the five folds: Smoking history, Histological type, D-D,FIB, AGR,CEA CYFRA21-1, NSE, SII, and SIRI. The stability of feature selection was evaluated via 100 bootstrap resampling iterations, demonstrating a mean inclusion frequency of 82.3% and a Jaccard similarity coefficient of 0.78 (**Supplementary Figures 2A–C****).** Multicollinearity diagnostics showed all variance inflation factors below 2.5, confirming the absence of significant collinearity among the selected predictors.

Using these ten features, we constructed and systematically compared nine machine learning models for baseline distant metastasis identification. Under the nested 5-fold cross-validation framework, all nine models were trained on the training partition of each fold and subsequently evaluated on the corresponding validation partition, with AUC recorded for each fold. By comparing the mean validation AUC across models, AdaBoost achieved the highest average validation AUC of 0.821 (SD = 0.033), outperforming NNET (0.778), RF (0.759), LGBM (0.753), LR (0.752), SVM (0.745), XGBoost (0.741), KNN (0.704), and DT (0.669), and was consequently selected as the optimal classifier (**Table 4**). Following algorithm selection via nested CV, we retrained all nine models on the full training set and compared their generalization performance on the independent test set. AdaBoost again achieved the best performance, with an AUC of 0.840 (95% CI: 0.770–0.910), a sensitivity of 71.7%, and a specificity of 71.4%, demonstrating robust generalizability. Compared with the LASSO-only strategy and the full feature model, which yielded AUCs of 0.826 and 0.815, respectively, our intersection approach achieved the highest test AUC of 0.840 and the smallest AUC variance, demonstrating favorable robustness and generalizability in internal validation (**Supplementary Figure 3D**; **Supplementary Table 2**). This triple-pronged strategy, combined with multicollinearity diagnostics, yielded ten core predictors with strong discriminative power and stability, effectively mitigating the bias inherent in any single selection algorithm. Calibration was assessed primarily via calibration plots with smoothed loess curves, supplemented by the mean absolute error (MAE) as a quantitative summary. The calibration curves demonstrated close agreement between predicted and observed probabilities in both the training and test sets, with MAE values of 0.048 and 0.057, respectively, indicating favorable calibration. The Hosmer–Lemeshow test yielded P-values of 0.053 and 0.122 for the training and test sets; however, given the well-recognized sample-size dependency of this test, these results were interpreted as supplementary reference rather than primary evidence of calibration adequacy. Decision curve analysis further revealed substantial net clinical benefit across a wide range of threshold probabilities (0.12–0.90 in the training set and 0.13–0.95 in the test set), underscoring its practical utility in identifying patients who may benefit from intensified staging workup. Our model is intended as a triage filter to guide resource allocation in the staging pathway, rather than as a replacement for imaging-based staging. Patients with high predicted risk, particularly those exceeding the recommended threshold of 0.65, would be candidates for prioritized or more advanced imaging, whereas those with low predicted risk might be considered for reevaluation of the intensity of their staging workup. However, given the current test sensitivity of approximately 72%, the model is not sufficiently accurate to rule out metastatic disease or to justify omitting standard staging investigations. To guide threshold selection, we evaluated sensitivity, specificity, and Youden’s index across thresholds from 0.55 to 0.75. We recommend 0.65 as the default reference threshold, at which sensitivity (71.7%) and specificity (71.4%) are optimally balanced, while 0.60 may serve as a “warning threshold” favoring higher sensitivity for screening purposes (**Supplementary Table 3**). It is important to emphasize that the Youden-derived threshold of 0.65 represents a statistical discrimination cut-off rather than a clinically validated decision threshold. To facilitate bedside application, we also constructed a nomogram based on the final model, enabling individualized risk scoring using the ten routine serum biomarkers and supporting stratified management strategies. The nomogram in this study was derived from a surrogate logistic regression fitted to predicted probabilities generated by AdaBoost. As AdaBoost captures complex non-linear patterns, the linear surrogate model cannot perfectly replicate all predictive signals of the ensemble model. As quantitatively verified in **Supplementary Table 5**, the surrogate model retained favorable discrimination (AUC = 0.901 vs 0.981 for AdaBoost) with high consistency in predicted risks (Pearson r = 0.869). Only a slight increase in Brier score and mild probability shrinkage (calibration slope = 0.876) were observed, indicating limited information loss introduced by linear approximation. The calibration consistency between the original AdaBoost model and the surrogate nomogram was further visualized in **Supplementary Figure 4**. This trade-off between interpretability and minor predictive decay should be considered when applying the nomogram in clinical practice.

When contextualized against the current TNM staging system and previously reported prediction models, the advantages of our approach become more apparent. First, although the ninth edition of the TNM classification (TNM-9) has refined N2 into single-station (N2a) and multi-station (N2b) involvement, and M1c into single-organ (M1c1) and multi-organ (M1c2) dissemination, it remains fundamentally anatomical in nature. External validation in 7,429 surgically resected NSCLC patients demonstrated a C-index of only 0.769 for TNM-9, a minimal improvement of 0.002 over the eighth edition (70). Prognostic heterogeneity persists even within the same stage: patients with T1N2a disease down-staged to IIB have significantly worse overall survival than those originally staged as IIB. Preoperative discrimination between N2a and N2b remains extremely challenging, and accurate differentiation requires invasive procedures such as EBUS-TBNA, limiting feasibility in resource-limited settings. Second, previously reported multi-marker models have been constrained by similar limitations. Some have been confined to a single histological type—for instance, the combination of D-dimer and fibrinogen achieved an AUC of only 0.802 for baseline metastasis in non-small cell lung cancer. Others have been limited to a single metastatic site, such as multi-marker panels for bone metastasis reaching an AUC of 0.955. Still others have shown limited predictive performance: the nomogram developed by Qi et al (66). based on 511 patients achieved a C-index of only 0.710–0.714, and the optimal machine learning model in Du et al.’s study of 1,629 lung cancer patients achieved a test AUC of only 0.810 (71). In contrast, our model integrates four dimensions and achieved an independent test AUC of 0.840, surpassing the reported values above and providing a biology-informed complement to anatomical TNM staging.

To assess whether the model’s performance was primarily driven by histological subtype differences rather than metastasis-specific biology, we conducted a series of subgroup and sensitivity analyses. In the NSCLC-only analysis excluding all SCLC patients (n = 348), the model maintained a test AUC of 0.803 (95% CI: 0.718–0.888), with only a modest reduction of 0.037 from the full model. When histological subtype alone was excluded from the full model, the AUC decreased minimally from 0.840 to 0.829; exclusion of smoking history yielded an AUC of 0.822; and simultaneous exclusion of both variables resulted in an AUC of 0.824 (**Supplementary Figure 3**; **Supplementary Table 4****).** These findings consistently demonstrate that the model’s predictive signal is predominantly driven by the integrated serum biomarkers—reflecting systemic inflammation, coagulation, nutritional status, and tumor burden—rather than relying on histological subtype or smoking history.

To enhance model interpretability, we applied the SHAP framework. SHAP analysis not only quantified the global contribution of each predictor but also enabled individualized explanation of model predictions, thereby substantially improving model transparency. The analysis identified smoking history, histological type, and key serological indicators—including D-dimer, fibrinogen, albumin-to-globulin ratio, carcinoembryonic antigen, CYFRA21-1, neuron-specific enolase, systemic immune-inflammation index, and systemic inflammation response index—as the features with the greatest predictive contributions to the model’s risk stratification output. Collectively, these results validate the robustness and clinical relevance of our modeling approach for baseline metastasis risk stratification and support its potential utility as a non-invasive triage tool in routine clinical practice.

## Conclusion

In this single-center, retrospective study, we developed and internally validated a pretreatment risk stratification model for baseline distant metastasis in lung cancer using the AdaBoost algorithm. By integrating readily accessible, multi-system laboratory indicators—reflecting systemic inflammation, coagulation status, tumor burden, and nutritional condition—and employing rigorous feature engineering alongside comparative machine learning analysis, the model demonstrated promising discriminative ability and satisfactory calibration. The incorporation of SHAP-based interpretability analysis further enhanced model transparency, facilitating the translation of algorithmic outputs into clinically interpretable risk estimates. As a low-cost and non-invasive tool based on routine laboratory tests, this model shows preliminary promise as an adjunctive aid for baseline metastasis risk stratification. However, we acknowledge that the current findings are derived from a single-center cohort with internal validation only; external validation in independent, ideally prospective, multicenter cohorts is warranted before any claim regarding clinical implementation or routine decision-making can be made.

### Limitation

Several limitations of this study warrant consideration. First, the single-center, retrospective design and the modest sample size precluded external or prospective validation, with all performance estimates derived from internal validation. Although we implemented a nested 5-fold cross-validation strategy in conjunction with an independent test set, both the training and test sets originated from the same institution, rendering all evaluations inherently internal. Internal validation alone cannot account for between-center heterogeneity in patient populations, laboratory assays, or clinical practices, nor can it substitute for external validation in establishing generalizability. Therefore, the current findings should be interpreted as exploratory, and the model’s performance in external populations or other clinical settings remains to be determined. Second, the model was developed to identify baseline distant metastasis at initial diagnosis rather than to predict future metastatic events, and its utility as a triage tool for selecting patients who may benefit from intensified staging workup requires prospective evaluation. Third, although we incorporated a broad panel of peripheral blood biomarkers, we did not include imaging-based radiomic features, circulating tumor cells, circulating tumor DNA, or molecular pathology data—all of which could potentially enhance predictive performance. Fourth, the model provides an estimate of overall metastasis risk without offering site-specific predictions for brain, bone, liver, or adrenal metastases, thereby limiting its utility for guiding targeted surveillance.

In summary, although the AdaBoost-based model demonstrated promising internal discriminative ability and calibration in this cohort, it is not yet ready for routine clinical application. Prospective, multicenter external validation in larger and more diverse populations remains imperative prior to any consideration of clinical translation.

## Data Availability

The dataset generated and analyzed during this study comprises retrospective patient records from Guangzhou First People’s Hospital, including clinical, laboratory, and imaging data. Due to the sensitive nature of patient health information and in compliance with the hospital’s institutional review board (IRB) policies and the Chinese regulations on data privacy, the full dataset is not publicly available. All data were anonymized and de-identified prior to analysis, and access is strictly restricted to the investigators who directly participated in this study. Data access is not available due to patient confidentiality and institutional ethical policies. No name, email, or website is provided for dataset requests because the data cannot be shared with external parties.
