# Supplementary material for: Development and validation of a machine-learning-based triage model incorporating systemic inflammatory, nutritional, coagulation, and tumor marker parameters for identifying baseline distant metastasis in lung cancer
Source: Front Oncol. 2026 Sep 8;16:1929804. doi: 10.3389/fonc.2026.1929804 (PMC13593466; doi:10.3389/fonc.2026.1929804)
Supplement: Supplementary file 1 [file DataSheet1.docx]

**Figure S1. ROC curves of six feature set models.** ROC curves for six AdaBoost models on the independent test set (n = 123). The full model (Model 1) achieved the highest AUC of 0.840 (95% CI: 0.767–0.912), while the inflammatory index‑only model (Model 6) performed worst (AUC = 0.682, 95% CI: 0.597–0.767). A stepwise improvement in performance was observed with the integration of more feature dimensions, supporting the synergistic value of multi‑dimensional biomarkers.

**Figure S2. Feature selection stability assessment based on 100 bootstrap resampling iterations and ROC curve comparison of three feature selection strategies. Figure S1‑A.Bootstrap feature selection frequency. Figure S1‑B.Distribution of cross‑validation AUC values from bootstrap samples. Figure S1‑C. Distribution of Jaccard similarity coefficients. Figure SX‑D. ROC curves of three feature selection strategies. Receiver operating characteristic curves comparing AdaBoost models using three feature selection strategies on the independent test set. Strategy A (our approach) selected 10 features via the intersection of LASSO, Boruta, and RFE; Strategy B selected 8 features via LASSO alone; Strategy C included all 24 candidate features. The corresponding test AUCs were 0.840, 0.826, and 0.815, respectively. Strategy A achieved the highest AUC and the most favorable sensitivity‑specificity trade‑off.**

**Figure S3. ROC curves of sensitivity analyses for histological subtype and smoking history.** Receiver operating characteristic curves for five AdaBoost models on the independent test set. Model A (full model, AUC = 0.840) represents the reference performance. Models C–E (excluding histological subtype, smoking history, or both) showed minimal AUC reductions (≤0.018). Model B (NSCLC‑only, excluding SCLC) maintained clinically meaningful performance (AUC = 0.803). These findings indicate that the model's predictive signal is primarily driven by serum biomarkers rather than histological subtype or smoking history. Abbreviations: ROC, receiver operating characteristic; AUC, area under the curve; NSCLC, non‑small cell lung cancer; SCLC, small cell lung cancer.

**Table S5.** **Quantitative consistency comparison between original AdaBoost and surrogate logistic regression model.**

|  | AdaBoost | Surrogate | Difference |
| --- | --- | --- | --- |
| AUC | 0.981 | 0.901 | 0.08 |
| Brier | 0.0359 | 0.0458 | 0.0099 |
| Pearson | NA | 0.869 | NA |
| Spearman | NA | 0.8581 | NA |
| Calibration_slope | NA | 0.876 | NA |

**Note:** **AUC, Brier score, correlation coefficients and calibration slope were applied to evaluate prediction and calibration agreement. High correlation reflected consistent risk ranking, while minor changes in AUC and Brier score indicated limited information loss caused by linear surrogate approximation.**

**Figure S4. Calibration curves for AdaBoost and surrogate logistic regression model.**

**Note:** **Solid line: AdaBoost; dashed 、 surrogate logistic regression; dotted line: ideal reference line. Slight deviation between curves represented mild discrepancy in calibration.**
